# Testing New Hypotheses Regarding Ebolavirus Reservoirs

**DOI:** 10.3390/v8020030

**Published:** 2016-01-26

**Authors:** Siv Aina Jensen Leendertz

**Affiliations:** Zaunkönigsteig 6A, 14621 Schönwalde-Glien, Germany; sivaina@hotmail.com; Tel.: +49-160-7573-418

**Keywords:** ebolavirus, Ebola virus, reservoir, filovirus ecology, origin, alternative hypotheses

## Abstract

Despite a relatively long search for the origin of ebolaviruses, their reservoirs remain elusive. Researchers might have to consider testing alternative hypotheses about how these viruses persist and emerge to advance ebolavirus research. This article aims to encourage researchers to bring forward such hypotheses, to discuss them scientifically and to open alternative research avenues regarding the origin and ecology of ebolaviruses.

The largest Ebola virus disease (EVD) outbreak that has ever been recorded is currently waning. However, the ecology of the causative agents of EVD (Bundibugyo virus (BDBV), Ebola virus, Sudan virus (SUDV), and Taï Forest virus (TAFV)) is still a mystery and future outbreaks cannot be predicted [1]. The current support for fruit bats as reservoirs for Ebola virus (EBOV) has been reviewed (e.g., [2,3,4]) and although accumulating data show that these bats, as well as insectivorous bats, may be intermittently exposed to filoviruses, it is questionable whether these viruses are circulating regularly in bat populations. Bats have longer life spans than most other mammals of similar size, can fly over great distances, and are able to clear filovirus infections. This means that seropositive specimens may be sampled for a relatively long time in areas geographically distant from where the specimens were exposed to ebolaviruses. Hence, the sampling area might not represent the original infection hot spot. Prediction of emergence risk areas relies heavily on the distribution of the suspected fruit bat reservoirs for EBOV (predominantly Franquet’s epauletted fruit bats (species *Epomops franqueti*), hammer-headed fruit bats (*Hypsignathus monstrosus*), and little collared fruit bats (*Myonycteris torquata*)). Consequently, the majority of the tropical belt of Africa is considered to be at risk for EVD outbreaks [5]. Critically, the geographical distribution of the fruit bats of these three species does not explain the emergence of genetically distinct ebolaviruses (BDBV, SUDV, TAFV) that have occurred across Africa, and the seemingly dead-end infection of experimentally infected bats does not support maintenance of virus circulation in these hosts [6]. Additionally, biologically plausible hypotheses about how ebolaviruses persist and emerge need to be tested. The purpose of this article is therefore to encourage an open exchange of such alternative hypotheses that might not be in line with current dogma nor represent the views held by the majority of virologists, and to catalyze subsequent cross-disciplinary research.

Excellent and enormous research efforts on bats have brought insight into filoviruses’ ecology [2,3]. Such research should surely continue but also expand into exploring the possibility of one or more “missing links” between bats and hypothesized true reservoirs. One could perhaps initiate a systematic re-assessment of ecological factors involved in ebolavirus emergence, even if for no other reason than to exclude them. It is perhaps incidental that there is a delineation of SUDV and EBOV by river basins (EVD outbreaks due to SUDV infection have so far occurred within the River Nile Basin only; EBOV spillovers in humans and possibly apes occurred on or near tributaries of the Congo River, Oogoué River and other rivers within the distribution of the original Guinean-Congolese rainforest) [5,7,8]. Nevertheless, such separation speaks against a single well-mixed population able to cross basin borders (such as fruit bats) serving as the reservoir for all ebolaviruses. Such separation of hosts by means of rivers draining tropical Africa in opposite directions could perhaps explain why genetically distinct ebolaviruses can emerge in places that are geographically very close [9]. It could also explain the occurrence of the possibly recombinant EBOV variants that were identified in great ape carcasses from the Gabon/Republic of Congo border area (a recombination of the strains occurring on either side of the border) [10], an area that is drained by opposing river basins. Apes, or intermediate hosts such as bats, perhaps had access to two different infective sources associated with the opposing river basins and hence acted as “viral melting pots”. Walsh and colleagues [11] investigated the great ape outbreaks in the forest areas and national parks in North East Gabon and Congo and suggested an on-land spread of the virus or reservoir guided by the river tributaries. Interestingly, their model including time, genetic change of virus, and geographic distance between outbreaks fit the data much better when the proposed transmission wave was routed via the branching point of two tributaries of the Ivindo River that connected the outbreak sites.

A potential connection between ebolaviruses and rivers is just one proposed hypothesis to call for alternative research avenues and substantial broadening of multi-disciplinary collaborations. Rivers have their own unique flora and fauna in the tropical ecosystem and many ecological aspects of rivers that might be important key events for viral emergence have not been considered. Further, it might be presumed by many that ebolaviruses are limited to terrestrial animal hosts. However, aquatic or semi-aquatic animals could potentially represent reservoir hosts and provide ebolaviruses with an ecological link from an original aquatic environment to the terrestrial environment. Of course, rivers are found anywhere in tropical Africa and, typically, human settlements occur along rivers. However, the possibility of viral persistence in hosts in or by rivers and river beds should not be disregarded as part of ebolavirus ecology without good reasons. Viral persistence in local riverine hosts might have been the cause of the so-far unexplained high genetic similarity between EBOV isolates from human outbreaks in Luebo, Democratic Republic of the Congo, that occurred in two consecutive years [12]. A systematic review of African waterways, their related fauna and hosts, as well as their use as transportation corridors for human and animals, may allow new perspectives on geographical, seasonal, and ecological patterns (e.g., optimal water flow, water level required to reach a particular sediment stratum, any local changes to the hydrology as irrigation and artificial dams, host movement). Such a review may provide alternative factors for mathematical modeling aimed at characterizing the ecological niche of ebolaviruses [5,13,14]. Such models should be stratified for each particular ebolavirus to accommodate for potential variation in ecological requirements between members of different ebolavirus species.

In line with the river hypothesis, riverine insects that are occasionally present in bats’ diets or are otherwise overlapping ecologically may be providing the virus with an ecological link from an original aquatic or semi-aquatic environment to terrestrial hosts. Virus may be concentrated or perhaps even activated through particular bat feeding and digestion processes. Early research indeed investigated the potential role of insects as hosts of EBOV, although few experimental infections were done and no ebolavirus was identified in the subset of insect diversity that was collected during post-outbreak screenings [4,15,16]. No sampling of aquatic insects in the nearby streams was done. It might have been assumed that a reservoir insect should be able to infect humans (or animals) through biting, or that the insect should spend most of its lifecycle on land or in the air. The possibility that non-biting, aquatic insects could be involved in ebolavirus ecology can be illustrated with the example of mayflies. Mayflies (order *Ephemoptera,* more than 3000 recognized species) are evolutionary ancient aquatic insects. They spend most of their lives as eggs or larvae in sediment and other material under water where they feed on biological material for up to two and a half years before they emerge from the aquatic habitat as adults, sometimes in large masses [17,18]. For some mayflies of some species, such mass emergence may cause Potomac horse fever when rickettsia-infected mayflies are ingested by horses that are grazing on the river bank [19]. This example shows that the ecological transmission of a virus between emerging infected mayflies and susceptible mammals is indeed possible. Adult mayflies live only for a few hours or days during which they do not feed. Hence, these and possibly other insects that do not parasitize humans or animals may therefore be overlooked as virus reservoirs as they are not truly “vectors”. The ephemeral life cycle of mayflies shows the possibility that a host and associated virus could be unavailable from susceptible mammal hosts for most of the insect’s life. Some animals that are feeding on mayfly larvae or adults, such as frogs, birds, and possibly fish, could be resistant to infection [16] and could perhaps act as natural buffers of viral emergence. A local reduction or depletion of such buffer animals due to natural or human-induced changes could affect a natural equilibrium and influence the chance of virus emergence.

The ecology of filoviruses other than ebolaviruses [20,21] is also not determined; for Marburg virus and Ravn virus, many findings favor Egyptian rousettes (*Rousettus aegypticus*) as natural reservoirs, although some important questions remain unanswered [2,3,4]. It appears that human infection with these viruses is associated with entry into caves at specific seasons that coincide with active infection of six-month-old rousettes [22,23]. However, no bat-to-bat transmission of Marburg/Ravn virus has been demonstrated and it is questionable if waning antibodies could explain the peak in infection around six months as the majority of pups are naïve from birth [22,24,25]. It needs to be addressed whether these infection peaks are truly a result of fluctuations in bat population susceptibility to infection or rather due to seasonally increased exposure to another virus source. As caves appear to be the focal site of human infection, the infection peaks may possibly be due to an influx of aquatic insects into caves during dry seasons when temporal water sources shrink. Perhaps the juvenile bats’ roosting position in the caves, *i.e.*, close to the cave mouth and relatively far down on the walls, overlaps with preferred spots for specific insects and predisposes them to infection.

The many “perhaps” and “possibly” in this article reflect the need to investigate a number of aspects of a potential complex filovirus ecology; it presents a unique challenge to unite researchers from a number of fields to synergistically discuss and explore new research avenues. The hypothesis that rivers are important for ebolavirus ecology may indeed prove wrong. However, such hypotheses may invite researches to take an ecological view and critically reconsider and review if there are other factors that influence filovirus disease outbreak patterns more than the general distribution of the various bats that so far have been implicated. EBOV, and other filoviruses, provides us with an opportunity to work on the One Health concept in the broadest sense by expanding multidisciplinary brainstorming to include experts in fields such as entomology, hydrology, geology, and riparian forest biology to discover factors and patterns that might not have previously been considered important for infectious disease emergence [26]. Importantly, a list of potential hypotheses to be tested will give on-site outbreak investigators a broader spectrum of ecological factors to record, additional epidemiological pathways to investigate, and a longer list of biological material to be collected.
